# Supplementary material for: Remimazolam-etomidate versus remimazolam-propofol for gastrointestinal endoscopy: A randomized controlled trial
Source: PLoS One. 2025 Jun 11;20(6):e0326043. doi: 10.1371/journal.pone.0326043 (PMC12157239; doi:10.1371/journal.pone.0326043)
Supplement: S1 Table — (DOCX) [file pone.0326043.s001.docx]

**S1 Table. Multivariable logistic regression for respiratory depression**

| Variables | Multivariable logistic regression | | | | |
| --- | --- | --- | --- | --- | --- |
|  | β | S.E | Z | *P* | OR (95% CI) |
| Group |  |  |  |  |  |
| RP |  |  |  |  | 1.00 (Reference) |
| RE | -1.04 | 0.40 | 6.75 | 0.009 | 0.35 (0.16-0.77) |
| Sex |  |  |  |  |  |
| Female |  |  |  |  | 1.00 (Reference) |
| Male | -0.67 | 0.57 | 1.38 | 0.240 | 0.51 (0.17-1.56) |
| Age | -0.40 | 0.22 | 3.36 | 0.067 | 0.96 (0.92-1.00) |
| ASA |  |  |  |  |  |
| III |  |  |  |  | 1.00 (Reference) |
| I-II | 0.746 | 1.13 | 0.44 | 0.507 | 2.11 (0.23-19.11) |
| BMI | 0.13 | 0.07 | 3.54 | 0.060 | 1.14 (0.99-1.30) |
| Comorbidities |  |  |  |  |  |
| Yes |  |  |  |  | 1.00 (Reference) |
| No | -1.25 | 0.44 | 7.95 | 0.005 | 0.29 (0.12-0.68) |
| Sleep apnea |  |  |  |  |  |
| Yes |  |  |  |  | 1.00 (Reference) |
| No | -2.56 | 0.54 | 22.78 | 0.000 | 0.08 (0.03-0.22) |
| PRODIGY | 0.10 | 0.06 | 2.43 | 0.119 | 1.11 (0.97-1.26) |

OR: Odds Ratio, CI: Confidence Interval, RP: Remimazolam-propofol, RE: Remimazolam-etomidate, ASA: American Society of Anesthesiologists, BMI: Body Mass Index
