# Supplementary material for: Remimazolam-etomidate versus remimazolam-propofol for gastrointestinal endoscopy: A randomized controlled trial
Source: PLoS One. 2025 Jun 11;20(6):e0326043. doi: 10.1371/journal.pone.0326043 (PMC12157239; doi:10.1371/journal.pone.0326043)
Supplement: S1 Protocol — (PDF) [file pone.0326043.s002.pdf]

Protocol

Project Name: Efficacy of remimazolam combined with etomidate or propofol for gastrointestinal endoscopy: a single-center, randomized controlled clinical trial

Protocol Version Number: 1.0

Protocol Version Date: December 20, 2023

Project Leader (Signature):

Date (Signature):

Department:

I. Research Background and Significance

Gastrointestinal endoscopy plays an irreplaceable role in the diagnosis and treatment of gastrointestinal diseases. With the continuous pursuit of comfortable medical care, the demand and requirements for painless gastrointestinal endoscopy are increasing. The commonly used sedative anesthetic drugs currently have significant inhibitory effects on hemodynamics and respiration, leading to a higher incidence of sedation-related adverse events [1]. Propofol and etomidate are currently commonly used drugs for sedation in gastrointestinal endoscopy, but both may cause adverse events such as hypoxemia, hypotension, myoclonus, and injection pain [2-3]. Remimazolam is a new type of benzodiazepine drug, characterized by rapid onset and rapid metabolism, with a lower incidence of hemodynamic and respiratory-related adverse events compared to propofol and etomidate [4-6]. However, remimazolam alone requires a higher dose to meet sedation/analgesia requirements, and under high-dose conditions, hemodynamic instability and respiratory depression can still occur. Optimizing drug use by combining the advantages and disadvantages of the three drugs is the main purpose of this study. Therefore, this study aims to evaluate the efficacy of remimazolam combined with etomidate or propofol for sedation/analgesia in painless gastrointestinal endoscopy, providing a safer and more optimal drug regimen for clinical use.

II. Research Objectives

To evaluate the clinical efficacy of remimazolam combined with etomidate or propofol for painless gastrointestinal endoscopy.

III. Research Design

1. Study Design

This study is a single-center, double-blind, randomized controlled clinical trial. Written informed consent must be obtained from participants before any trial procedures are conducted.

2. Study Population

Inclusion Criteria:

1. Age > 18 years, scheduled for gastrointestinal endoscopy under procedural sedation;
2. ASA I-III.

Exclusion Criteria:

1. Refusal to sign the informed consent;
2. Severe respiratory depression;
3. Acute or severe bronchial asthma;
4. Known or suspected gastrointestinal obstruction;
5. Known allergy to any of the drugs or components used in this study;
6. Severe liver or kidney dysfunction;
7. Long-term use of opioid drugs;
8. Long-term use of benzodiazepines;
9. Anticipated difficult airway;
10. Adrenal insufficiency.

Exclusion Criteria During the Study:

1. Unexpected events during sedation/analgesia or surgery;
2. Participants who withdraw from the study on their own;
3. Loss to follow-up.

3. Randomization and Blinding

Researchers will use the website (www.random.org) to generate a block randomization scheme. All participants will be randomly assigned to receive remimazolam combined with etomidate or propofol for sedation/analgesia in a 1:1 ratio. The participants, anesthesiologists, endoscopists, and postoperative evaluators will be blinded to the group assignments. The anesthesiologist will be responsible for intraoperative and postoperative assessments. The group assignments will not be blinded to the anesthesia nursing staff who prepare the anesthetic drugs.

4. Study Interventions

Before sedation/analgesia for gastrointestinal endoscopy, all participants will undergo preoperative evaluation at the anesthesia clinic. Baseline data will be collected, including age, gender, height, weight, ASA classification, Mallampati classification, drinking history, surgical history, comorbidities (hypertension, diabetes, cerebrovascular disease, cardiovascular disease, liver and kidney diseases), and PRODIGY score [7]. Patients will be fasting for 6 hours and no fluids for 2 hours before the procedure. Upon entering the operating room, patients will be positioned according to the type of examination, and monitoring devices will be connected to record baseline vital signs

(non-invasive blood pressure, oxygen saturation, ECG, end-tidal carbon dioxide). Preoxygenation with an endoscopic mask (8-10 L/min, 3-5 min) will be performed under spontaneous breathing, and intravenous access will be established. Resuscitation drugs such as epinephrine, atropine, ephedrine, and phenylephrine will be prepared.

The anesthesia nursing staff will prepare the sedation/analgesia drugs according to the group assignment. Remimazolam tosilate (RT) will be diluted with normal saline to 1.25 mg/mL, and individualized dosing will be administered based on patient weight. All patients will receive lidocaine gel for pharyngeal surface anesthesia before undergoing gastroscopy. Oxygen will be administered via an endoscopic mask at 6 L/min from the start of the endoscopic procedure until the patient is fully awake. According to the expert consensus on sedation/analgesia for gastrointestinal endoscopy, remimazolam will be administered as a single intravenous (IV) bolus over 1 minute using a micro-infusion pump. The initial loading dose of remimazolam will be 0.15 mg/kg, combined with etomidate 0.1 mg/kg or propofol 0.75 mg/kg, and sufentanil 5-10 mcg. The analgesic drug will be administered 3 minutes before the sedative drug. When the patient is sufficiently sedated (Modified Observer's Assessment of Alertness/Sedation [MOAA/S] ≤ 3), the endoscopic examination will commence. If sedation is deemed inadequate or intubation fails, up to 5 supplemental doses (RT 2.5 mg) of intravenous bolus can be administered at 1-minute intervals within 15 minutes after the initial dose. If the initial dose and supplemental doses are insufficient to achieve adequate sedation for endoscopic insertion, the anesthesiologist will decide whether to administer a rescue sedative drug (propofol 0.75 mg/kg) at the start of the procedure. Once the procedure begins, to maintain an adequate level of sedation throughout the examination (MOAA/S score ≤ 3), supplemental doses of remimazolam will be administered to the patient at least 1-minute intervals (not exceeding 5 supplemental doses in total) based on the anesthesiologist's judgment. If these doses are insufficient to maintain appropriate sedation, a rescue sedative drug will be administered to complete the examination. If the use of rescue drugs raises safety concerns, the examination can be completed under general anesthesia with endotracheal intubation.

Throughout the procedure, the level of sedation will be assessed using the MOAA/S score. The anesthesiologist, who is blinded to the group assignment, will be responsible for the MOAA/S scoring. Before enrolling patients in this study, the evaluating physician will complete at least one training session to standardize the MOAA/S scoring. The MOAA/S score will be recorded at the

start of the initial drug administration, and frequently during the procedure until the patient is fully awake (three consecutive MOAA/S scores of 5).

5. Observation Indicators

Primary Outcome:

The primary outcome is the incidence of respiratory depression (RD). Respiratory depression is defined as a respiratory rate < 8 breaths per minute and/or SPO2 < 90% [7]. Respiratory rate will be monitored using end-tidal carbon dioxide monitoring. When a decrease in respiratory rate or oxygen saturation occurs, the anesthesiologist will need to assess the accuracy of the values to ensure normal readings. If airway obstruction due to tongue base retraction is relieved by airway maneuvers and the respiratory rate is above the assessment threshold, it will not be judged as respiratory depression, but the number of airway maneuvers will be recorded.

Secondary Outcomes:

Secondary outcomes mainly include the incidence of intraoperative hypoxemia (SPO2 < 90%, > 10s), the number of minor airway maneuvers (such as jaw lift, patient repositioning, mask ventilation), the lowest SpO2, the number of times the endoscope is removed due to persistent hypoxemia, and the success rate of sedation/analgesia (i. Completion of the entire endoscopic procedure; ii. No need for alternative and/or rescue sedatives; iii. Up to 5 supplemental doses administered within 15 minutes after the initial dose).

The examination time (from intubation to removal of the endoscope), sedation/analgesia time (from administration of analgesic drugs to full awakening), and awakening time (from administration of flumazenil to full awakening) will be recorded. The number of sedative drug supplements, the dose of sedative drugs used, and the dose of analgesic drugs used will be recorded. Vital signs (MAP, HR, SPO2) at different time points will be recorded. After the patient is fully awake, the occurrence of nausea and vomiting will be recorded. After the examination, the endoscopist's satisfaction will be recorded. The endoscopist will subjectively evaluate based on the operating conditions and whether the operation was interrupted, with a full mark of 10 points, 0-3 points for dissatisfaction, 4-7 points for relative satisfaction, and 8-10 points for satisfaction.

On the first postoperative day (postoperative Day 1, POD1), patient satisfaction will be recorded. Patients will subjectively evaluate based on their own feelings. Similarly, the full mark is 10 points,

with 0-3 points indicating dissatisfaction, 4-7 points indicating relative satisfaction, and 8-10 points indicating satisfaction. The occurrence of nausea and vomiting, itching, and urinary retention will be recorded.

IV. Adverse Events

During the entire study period, any AEs will be recorded, including but not limited to infectious diseases, hematological tests, systemic diseases, and injection site pain. As for AEs, hypotension is defined as a decrease of 30% or more in SBP from the baseline value and/or a mean arterial pressure< 60 mmHg; hypertension is defined as an increase of 30% or more in SBP from the baseline value; bradycardia is defined as HR<60 beats per minute,>10s; tachycardia is defined as HR>100 beats per minute,>10s; respiratory depression is defined as RR< 8 breaths per minute, SPO2 <90%. Anesthesiologists will actively manage any abnormal conditions that occur in patients, including symptomatic treatment and active management of the underlying causes.

V. Statistical Analysis

1. Sample Size Calculation

Based on the clinical practice of this research center and previously published studies [9-10], the incidence of respiratory depression during gastrointestinal endoscopy with remimazolam tosilate combined with propofol is referenced at 30%. If it is expected that remimazolam combined with etomidate can reduce the incidence of respiratory depression by 50%, the single-group sample size calculated using PASS software is 118 (α=0.05, β=0.8). Considering a dropout rate of 5%, the single-group sample size is 124.

2. Statistical Analysis

Statistical analysis will be performed using SPSS software. Continuous variables will be presented as mean ± standard deviation (SD) or median M (P25, P75). The normality of continuous variable data will be assessed using the Kolmogorov-Smirnov test. Continuous variables that follow a normal distribution will be analyzed using the two-independent-samples t-test, while those that do not follow a normal distribution will be analyzed using the Mann-Whitney U test. Categorical variables will be expressed as counts and frequencies, and will be analyzed using the $\chi 2$ test or Fisher's exact test. All statistical tests will be two-sided with a significance level set at $P < 0.05$, and 95% confidence intervals (95% CI) will be calculated.

VI. Research Ethics

1. Ethics Committee Review

The protocol, informed consent form, and other materials directly related to the participants must be submitted to the ethics committee for review. The study can only be officially launched after obtaining written approval from the ethics committee. Researchers will conduct the study in accordance with the approved protocol and submit regular progress reports and reports of serious adverse events that occur during the study to the ethics committee as required. A final report will be submitted upon study termination or completion. Any changes to the protocol or informed consent form must be reported to the ethics committee in a timely manner. These changes should not be implemented until approved by the ethics committee, unless they are necessary to eliminate apparent and immediate risks to participants.

2. Informed Consent

Participants will not be enrolled in this study until they have signed the written informed consent form. If any changes to the informed consent form occur during the study, participants will be provided with and asked to sign the updated version in accordance with ethical requirements.

VII. Publication Plan and Participant Information Protection Measures

The results of this study may be published in medical journals. We will keep patient information confidential as required by law. Personal information of patients will not be disclosed unless required by relevant laws. When necessary, government regulatory authorities and the hospital ethics committee and its relevant personnel may review patient records in accordance with regulations.

VIII. References

[1] Razavi F, Gross S, Katz S. Endoscopy in the elderly: risks, benefits, and yield of common endoscopic procedures. Clin Geriatr Med. 2014 Feb;30(1):133-47.

[2] Wang D, Wang S, Chen J, et al. Propofol combined with traditional sedative agents versus propofol- alone sedation for gastrointestinal endoscopy: a meta-analysis. Scand J Gastroenterol. 2013 Jan;48(1):101-10.

[3] Hüter L, Schreiber T, Gugel M et al. Low-dose intravenous midazolam reduces etomidate-induced myoclonus: a prospective, randomized study in patients undergoing elective cardioversion.

Anesth Analg. 2007 Nov;105(5):1298-302, table of contents.

[4] Guo J, Qian Y, Zhang X et al. Remimazolam tosilate compared with propofol for gastrointestinal endoscopy in elderly patients: a prospective, randomized and controlled study. BMC Anesthesiol. 2022 Jun 10;22(1):180.

[5] Liu X, Ding B, Shi F et al. The Efficacy and Safety of Remimazolam Tosilate versus Etomidate-Propofol in Elderly Outpatients Undergoing Colonoscopy: A Prospective, Randomized, Single-Blind, Non-Inferiority Trial. Drug Des Devel Ther. 2021 Nov 16;15: 4675-4685.

[6] Hu Sun, Tao Wang, Xinzhi Xu, et al. Effective dose and adverse reactions analysis of Remimazolam for sedation in elderly patients undergoing gastroscopy [J]. National Medical Journal of China,2022,102(5):332-335.

[7] Khanna AK, Bergese SD, et al, PRediction of Opioid-induced respiratory Depression In patients monitored by capnoGraphY (PRODIGY) Group Collaborators. Prediction of Opioid-Induced Respiratory Depression on Inpatient Wards Using Continuous Capnography and Oximetry: An International Prospective, Observational Trial. Anesth Analg. 2020 Oct;131(4):1012-1024.

[8] Chen SH, Yuan TM, Zhang J, et al. Remimazolam tosilate in upper gastrointestinal endoscopy: A multicenter, randomized, non-inferiority, phase III trial. J Gastroenterol Hepatol. 2021 Feb;36(2):474-481.

[9] Jianwen Zhang, Zhihu Yang, Mingcui Qu, et al. Efficacy of low-dose remimazolam combined with etomidate or propofol for gastrointestinal endoscopy in the elderly patients [J]. World Clinical Drug,2023,44(07):734-739.

[10] Zhao MJ, Hu HF, Li XL, et al. The safety and efficacy between remimazolam and propofol in intravenous anaesthesia of endoscopy operation: a systematic review and meta-analysis. Int J Surg. 2023 Aug 3.
