# Supplementary material for: Remimazolam-etomidate versus remimazolam-propofol for gastrointestinal endoscopy: A randomized controlled trial
Source: PLoS One. 2025 Jun 11;20(6):e0326043. doi: 10.1371/journal.pone.0326043 (PMC12157239; doi:10.1371/journal.pone.0326043)
Supplement: S2 Protocol — (PDF) [file pone.0326043.s003.pdf]

研究方案

项目名称（中文）：瑞马唑仑复合依托咪酯或丙泊酚用于胃肠镜检查的临床效果观察：一项单中心、随机对照临床试验

项目名称（英文）：Clinical effects of remimazolam combined with etomidate or propofol for gastroenteroscopy: a single-center, randomized controlled clinical trial

研究方案版本号：1.0

研究方案版本日期：2023 年 12 月 20 日

项目负责人（签署）：

日　期（签署）：

科　室：

一、研究背景及意义

消化道内镜检查在消化道疾病诊疗中有不可替代的作用，随着人们对舒适化医疗的不断追求，对无痛消化内镜检查的需求和要求越来越高。目前常用的镇静麻醉药物对血流动力学和呼吸有较大的抑制作用，导致镇静相关不良事件的发生率较高[1]。丙泊酚和依托咪酯是目前临床上常用于胃肠镜镇静的药物，但二者可能会引起低氧血症、低血压、肌颤及注射痛等不良事件[2-3]。瑞马唑仑是一种新型的苯二氮草类药物，具有起效快、代谢迅速的特点，血流动力学及呼吸相关不良事件发生率低于丙泊酚和依托咪酯[4-6]。单用瑞马唑仑需要较高剂量来满足镇静/麻醉要求，在高剂量的背景下，仍会发生血流动力学不稳定和呼吸抑制。结合三种药物的优缺点从而更好的优化用药是本研究的主要目的。那么，本研究拟评价瑞马唑仑复合依托咪酯或丙泊酚用于无痛胃肠镜检查镇静/麻醉中的效果，为临床安全提供更优用药方案。

二、研究目的

评价瑞马唑仑复合依托咪酯或丙泊酚用于无痛胃肠镜检查的临床效果。

三、研究设计

1. 研究设计

本研究是一项单中心、双盲、随机对照临床试验。在开始任何试验方案规定的程序之前，需要获得参与者的书面知情同意书。

2. 研究人群

纳入标准：

（1）年龄>18 岁，择期于程序性镇静下行胃肠镜检查；(2) ASA I-III。

排除标准：

（1）拒绝签署知情同意书；（2）严重的呼吸抑制；（3）急性或重度支气管哮喘；（4）已知或可疑的胃肠道梗阻；（5）已知对本研究使用的药物及成分过敏；（6）严重肝、肾功能不全；（7）长期服用阿片类药物；（8）长期服用苯二氮卓类药物；（9）预期困难气道；（10）肾上腺皮质功能不全。

剔除标准：

（1）镇静/麻醉或手术过程出现意外；（2）研究过程中自行退出研究者；（3）失访。

3. 随机化和盲法

研究人员使用网站（www.random.org）生成区组随机化方案。所有纳入的参与者按照 1:1 的比例被随机分配到接受瑞马唑仑复合依托咪酯或瑞马唑仑进行镇静/麻醉。分组对参与者、麻醉医师、内镜医师、术后评估人员设盲，麻醉医师负责进行术中、术后评估。分组对准备麻醉药物的麻醉护理人员不进行设盲。

4. 研究干预措施

在进行胃肠镜诊疗镇静/麻醉前，所有参与者需在麻醉门诊进行术前评估。收集基线资料，包括年龄、性别、身高、体重、ASA 分级、Mallampti 分级、饮酒史、手术史、共病（高血压、糖尿病、脑血管病、心血管病、肝肾疾病）、PRODIGY 评分[7]。术前禁食 6 h，术前禁饮 2 h。患者入室，根据检查类别摆放体位，连接监护设备，记录基线生命体征（无创血压、血氧饱和度、ECG、呼吸末二氧化碳）。自主呼吸下使用内窥镜面罩充分给氧去氮（8-10 L/min，3-5 min），开放静脉通路。准备抢救药物肾上腺素、阿托品、麻黄碱、去氧肾上腺素。

麻醉护理人员根据分组准备镇静/麻醉药物，苯磺酸瑞马唑仑（Remimazolam tosilate，RT）使用生理盐水稀释至 1.25 mg/mL，根据患者体重进行个体化用药。所有患者在进行胃镜检查前服用利多卡因胶浆进行咽部表面麻醉。内镜操作期间至患者完全清醒期间使用内窥镜面罩给予吸氧 6 L/min。根据消化内镜镇静/麻醉诊疗专家共识，使用微量注射泵注在 1 分钟内进行单次静脉（IV）推注苯磺酸瑞马唑仑。使用瑞马唑仑初始负荷剂量为 0.15 mg/kg，复合依托咪酯 0.1 mg/kg 或丙泊酚 0.75 mg/kg，舒芬太尼为 50-100 mcg。镇痛药物于镇静药物推注前 3 min 给予。当患者充分镇静（Modified Observer's Assessment of Alertness/Sedation [MOAA/S] ≤ 3）时，开始消化内镜检查。如果认为镇静不充分或进镜失败，则在初始剂量结束后 1 分钟间隔允许 15 min 内最多 5 次补充剂量（RT 2.5 mg）静脉推注给药。如果初始剂量和补充剂量不足以获得内窥镜插入的充分镇静，则由麻醉医师决定在手术开始时给予镇静补救药物（丙泊酚 0.75 mg/kg）。一旦手术开始，为了使患者在整个检查过程中保持足够的镇静水平（MOAA/S 评分≤ 3），根据麻醉医师的判断，至少间隔 1 分钟向患者注射补充剂量（累计不超过 5 次补充剂量）的瑞马唑仑。如果这些剂量不足以维持适当的镇静，则给予镇静补救药物以完成检查。如果补救药物的使用引起安全性担忧，可进行气管插管全身麻醉

下完成检查。在整个手术过程中，使用 MOAA/S 评分评估镇静水平。盲态评估麻醉医师负责 MOAA/S 评分。在患者入组本研究之前，评估医生至少完成一次培训课程，以标准化 MOAA/S 评分。在首次给药开始时首先记录 MOAA/S 评分，在检查过程中，频繁记录 MOAA/S 评分，直至患者完全苏醒（连续 3 次 MOAA/S 评分为 5 分）。

5. 观察指标

2.5.1 主要结局（Primary outcome）

主要结局为呼吸抑制（respiratory depression，RD）的发生率。呼吸抑制定义为呼吸频率（respiratory rate）<8 次/分和/或 SPO_2<90%[7]。使用呼吸末二氧化碳监测呼吸频率，当出现呼吸频率或者血氧饱和度下降时，麻醉医师需要评估数值准确性，以确保读数正常。因舌体后坠，患者无效呼吸动作可通过气道操作解除气道梗阻，此时呼吸频率如果大于评估界值则不判断为呼吸抑制，但需要记录气道操作次数。

2.5.2 次要结局

次要结局主要包括术中低氧血症（SPO_2<90%，>10s）的发生率、需要进行轻微气道操作（如下颌抬起、调整患者姿势、面罩通气）的次数、最低 SpO2、因持续低氧血症取出内窥镜的次数、镇静/麻醉成功率（i 完成整个内窥镜检查程序; ii 不需要替代和/或急救镇静剂; iii 在初始剂量后 15 分钟内给予最多 5 次补充剂量）。

记录检查时间（进镜至退出内窥镜）、镇静/麻醉时间（给予镇痛药物至完全苏醒）、苏醒时间（给予氟马西尼至完全苏醒）。记录镇静药物补充次数、镇静药物使用剂量、镇痛药物使用剂量。记录不同时间点的生命体征（MAP、HR、SPO_2、）。

患者完全苏醒后记录患者恶心呕吐情况。

检查完成后，记录内镜医师满意度。内镜医师根据操作条件、操作是否中断等给予主观评价，满分 10 分，0-3 分为不满意、4-7 分为相对满意、8-10 分为满意。

术后第一天（postoperative Day 1，POD1），记录患者满意度。患者根据自身感受给予主观评价。同样，满分 10 分，0-3 分为不满意、4-7 分为相对满意、8-10 分为满意。记录患者恶心和呕吐、瘙痒、尿潴留发生情况。

四、不良事件

在整个研究期间，记录任何 AEs，包括但不限于感染性疾病、血液学检查、全身性疾病、注射部位疼痛等。作为 AEs，低血压定义为 SBP 较基线值下降 20%或以上和/或平均动脉压< 60 mmHg；高血压定义为 SBP 较基线值上升 20%或以上;心动过缓定义为 HR<60 次/分，>10s；心动过速定义为 HR>100 次/分，>10s；呼吸抑制定义为 RR< 8 次/分，SPO_2<90%。麻醉医师积极处理患者出现的异常情况，包括对症处理和积极处理病因。

五、统计分析

1. 样本量计算

综合本研究中心临床实践和既往发表研究[9-10]，苯磺酸瑞马唑仑复合丙泊酚用于胃肠镜检查时的呼吸抑制发生率以 30%作为参考，如若期许瑞马唑仑复合依托咪酯能够降低 50%的呼吸抑制发生率，通过使用 PASS 软件计算可得单组样本量为 118（α=0.05，β=0.8）。考虑到 5%的脱落率，单组样本量为 124。

2. 统计分析

使用 SPSS 软件进行统计学分析。连续变量以平均值±标准差（SD）或中位数 M（P25，P75）表示。使用 Kolmogorov-Smirnov 检验检查连续变量数据的正态分布。符合正态分布的连续变量使用两独立样本 T 检验，不符合正态分布的连续变量使用 Mann-Whitney U 检验。分类变量以数量和频率表示，使用 $\chi 2$ 检验、Fisher’s 精确检验进行处理。数据均采用双侧统计学检验，显著性设置为 $P<0.05$，并计算 95%置信区间（95% CI）。

六、研究相关伦理学

1. 伦理委员会审查

本方案和知情同意书及其他与受试者直接相关的资料必须提交伦理委员会，获得伦理委员会书面批准后方可正式开展研究。研究者将按照伦理委员会批准方案进行研究，按照要求定期向伦理委员会提交研究进展报告及研究中发生的严重不良事件。在研究中止和/或完成时，提交结题报告；及时向伦理委员会报告研究

方案或知情同意书等的变动情况，在未获得伦理委员会批准之前不得执行这些变动，除非是为了消除对受试者明显且直接的风险而做出的变更。

2. 知情同意

本项研究，在受试者签署书面知情同意书之前，受试者不得入组。在受试者参与期间，如发生知情同意书的变更，将按照伦理要求向受试者提供并签署更新版本。

七、发表计划及受试者信息保护措施

本项研究的结果可能会在医学杂志上发表，我们会按照法律要求为患者的信息保密，除非应相关法律要求，患者的个人信息不会被泄露。必要时，政府管理部门和医院伦理委员会及其有关人员可以按规定查阅患者的资料。

八、参考文献

[1] Razavi F, Gross S, Katz S. Endoscopy in the elderly: risks, benefits, and yield of common endoscopic procedures. Clin Geriatr Med. 2014 Feb;30(1):133-47.

[2] Wang D, Wang S, Chen J, et al. Propofol combined with traditional sedative agents versus propofol- alone sedation for gastrointestinal endoscopy: a meta-analysis. Scand J Gastroenterol. 2013 Jan;48(1):101-10.

[3] Hüter L, Schreiber T, Gugel M et al. Low-dose intravenous midazolam reduces etomidate-induced myoclonus: a prospective, randomized study in patients undergoing elective cardioversion. Anesth Analg. 2007 Nov;105(5):1298-302, table of contents.

[4] Guo J, Qian Y, Zhang X et al. Remimazolam tosilate compared with propofol for gastrointestinal endoscopy in elderly patients: a prospective, randomized and controlled study. BMC Anesthesiol. 2022 Jun 10;22(1):180.

[5] Liu X, Ding B, Shi F et al. The Efficacy and Safety of Remimazolam Tosilate versus Etomidate-Propofol in Elderly Outpatients Undergoing Colonoscopy: A Prospective, Randomized, Single-Blind, Non-Inferiority Trial. Drug Des Devel Ther. 2021 Nov 16;15: 4675-4685.

[6] 孙虎,王涛,徐志新,等. 瑞马唑仑用于老年患者胃镜检查镇静的有效剂量及不良反应分析[J]. 中华医学杂志,2022,102(5):332-335.

[7] Khanna AK, Bergese SD, et al, PRediction of Opioid-induced respiratory Depression In patients monitored by capnoGraphY (PRODIGY) Group Collaborators.

Prediction of Opioid-Induced Respiratory Depression on Inpatient Wards Using Continuous Capnography and Oximetry: An International Prospective, Observational Trial. Anesth Analg. 2020 Oct;131(4):1012-1024.
[8] Chen SH, Yuan TM, Zhang J, et al. Remimazolam tosilate in upper gastrointestinal endoscopy: A multicenter, randomized, non-inferiority, phase III trial. J Gastroenterol Hepatol. 2021 Feb;36(2):474-481.
[9] 张建文,杨智虎,渠明翠等.小剂量瑞马唑仑复合依托咪酯或丙泊酚用于老年人胃肠镜检查的效果观察[J].世界临床药物,2023,44(07):734-739.
[10] Zhao MJ, Hu HF, Li XL, et al. The safety and efficacy between remimazolam and propofol in intravenous anaesthesia of endoscopy operation: a systematic review and meta-analysis. Int J Surg. 2023 Aug 3.
